# RELIABILITY OF REMOTE SELF-ADMINISTRATED DIGITAL COGNITIVE ASSESSMENTS

**DOI:** 10.1093/geroni/igae098.4066

**Published:** 2024-12-31

**Authors:** Duong Huynh, Mary Patterson, Bin Huang

**Affiliations:** BrainCheck, Austin, Texas, United States; BrainCheck, Austin, Texas, United States; BrainCheck, Austin, Texas, United States

## Abstract

Early diagnosis of cognitive impairment and dementia relies on comprehensive, evidence-based cognitive assessment, which currently requires a clinic visit and access to skilled healthcare providers. This poses a challenge for people who live in areas with inadequate primary care services and those who have economic, insurance, or other transient hardships (transportation, time, etc.) that limit their access to healthcare services. Digital cognitive assessments (DCAs) with remote testing capabilities have emerged as an efficient and cost-effective solution. The aim of this study was to validate the reliability of BrainCheck, a platform for DCAs, when self-administered remotely. A total of 46 participants (60.9% female; age range 52-76) remotely completed a battery of eight BrainCheck cognitive assessments twice on the same device (iPad=8, iPhone=5, laptop=33): the participants self-administered in one session and were administered by a research coordinator (examiner) in the other session. The inter-session interval (ISI) varied across participants, from within the same day to 21 days apart. Testing outcomes, including the duration of time needed to complete the battery, the raw score from each assessment, and the raw overall score, were compared between the two sessions. We found moderate or good agreement between self- and examiner-administered performance, with intraclass correlation ranging from 0.59 to 0.83. Results from mixed-effects modeling further confirm the non-significant difference between self- vs. examiner-administered testing performance, which is independent of other factors including testing order, ISI, device, and participants’ demographic characteristics. These results demonstrate the feasibility of remote self-administration using BrainCheck.

